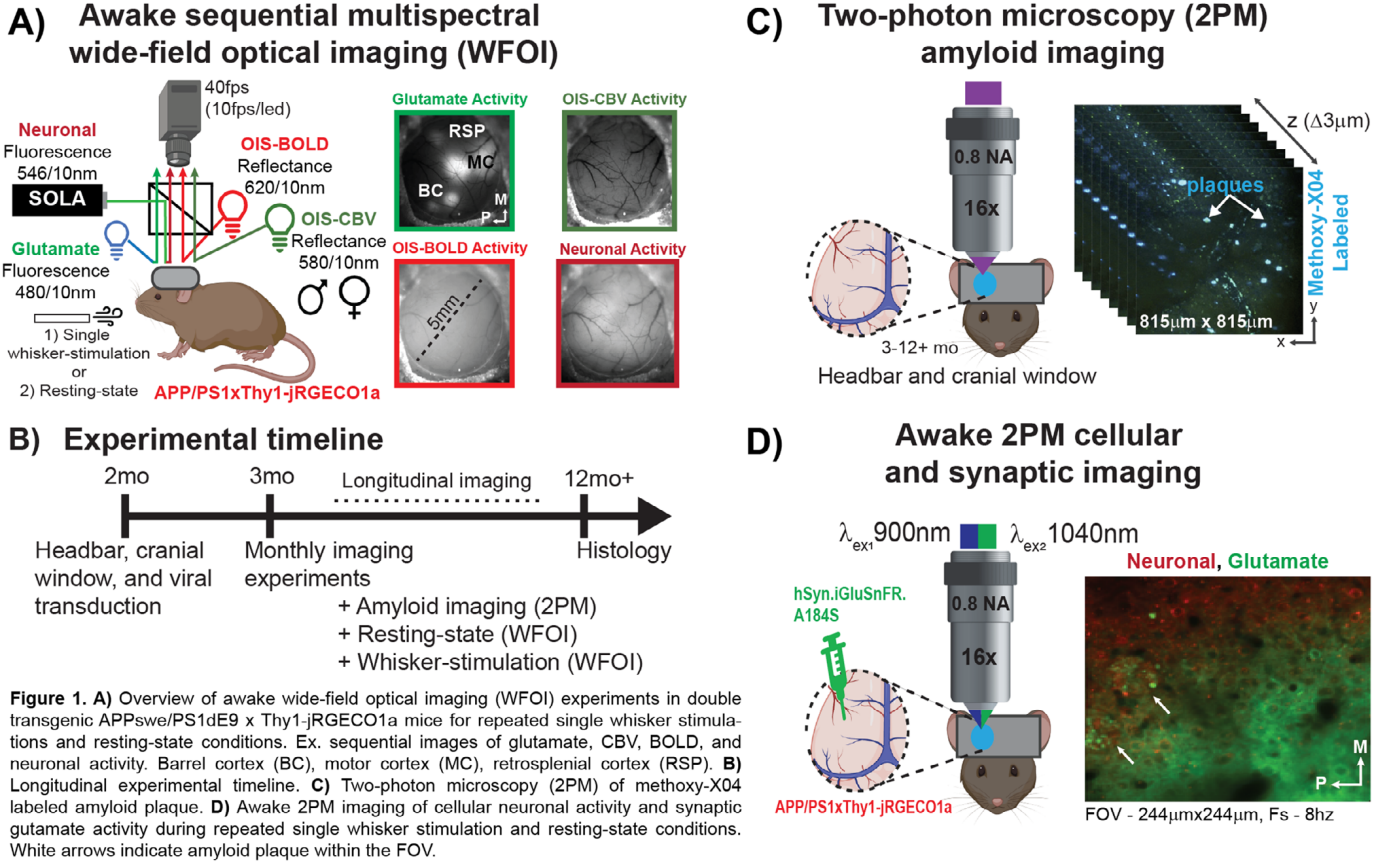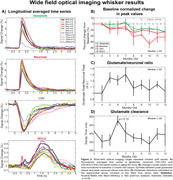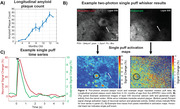# Longitudinal assessment of neuronal calcium, glutamate, and hemodynamic changes in aging APP‐PS1 mice

**DOI:** 10.1002/alz.091643

**Published:** 2025-01-09

**Authors:** Christopher G Cover, Jenna M Peretin, Alexander Poplawsky, Alberto L Vazquez

**Affiliations:** ^1^ University of Pittsburgh, Pittsburgh, PA USA; ^2^ University of Pittsburgh Department of Radiology, Pittsburgh, PA USA

## Abstract

**Background:**

Preclinical Alzheimer’s disease research has gained traction as a potential point of intervention, though it is relatively unknown how early stages of the disease impact cortical health. The following study utilizes optical imaging methods (Figure 1) to characterize changes in neuronal, glutamate, and hemodynamic activities in a preclinical amyloidosis mouse model of the disease.

**Method:**

Five (n = 5; 2 females & 3 males) APPswe/PS1dE9 x Thy1‐jRGECO1a double transgenic mice were breed for whole‐brain fluorescent imaging of neuronal activity. At two months of age, mice were injected with a fluorescent glutamate sensor in the right barrel cortex (BC), primary motor cortex (PMC) and retrosplenial cortex (RSP), then implanted with a 5mm cranial window. From 3‐12 months of age, mice were imaged monthly in the awake state using both wide‐field optical and two‐photon microscopy (2PM) to investigate both systems‐level and cellular/synaptic activities, respectively. Imaging sessions consisted of spontaneous resting‐state activity and evoked responses from a single, repeated whisker puff to the left whisker pad. Nonparametric statistical analysis was performed due to the small sample size.

**Result:**

Peak amplitudes progressively declined from baseline levels (i.e., 3 months of age) at the systems level in the aging mice and reached significance by 10 months of age (p < 0.05; Figure 2B). Additionally, a trend towards excitatory imbalances were seen from 5‐7 months of age. Interestingly, impairments in glutamate clearance trended towards significance only at 6 months of age, but not at other times. See Figure 3 for example 2PM in response to a single whisker puff.

**Conclusion:**

Longitudinal decline in stimulus‐evoked neuronal calcium, glutamate, and hemodynamic activities were seen in young, aging AD mice. Future directions will consist of 1) increasing the sample size, 2) analysis of resting‐state data at the systems and cellular levels, and 3) analysis of 2PM data with the whisker stimulus.